# On the Use of Pepsine Wine in the Artificial Feeding of Infants

**Published:** 1872-12

**Authors:** W. J. Cummins


### SELECTIONS.

---

### ON THE USE OF PEPSINE WINE IN THE ARTIFICIAL FEEDING OF INFANTS.

By W. J. CUMMINS, M D.

* * * * There is nothing, of course, like a good breast of milk for an infant, if it can be had; and in "the good old times," when the peasantry and small farmers lived on potatoes and milk, without stimulating their nerves with strong tea) nor their brains with penny-a-liner's words, there was an ample field for the selection of a foster-parent; but now even when the *rara avis*, a good nurse, is procured, she is so independent and knows her power so well, that any caprice must be humored, and she is always ready to throw up her situation or neglect her charge. A wet-nurse is, then, an admitted torment, and a balance struck between its advantage and disadvantage is generally against the former.

Artificial feeding by bottle is a great improvement upon the old system of spoon-feeding, as the act of suckling stimulates the salivary glands and insures due in-salivation, which is an important part of infantile digestion. With such an aid the stomach of most *human* infants is vigorous enough to fall into the way of digesting *cow's* milk, properly diluted, and mixed with sugar and cream to assimilate the proportions of its constituents to human milk—but besides the relative excess of casein and albumen contained in cow's milk when compared with human, the coagulum of the latter is " soft, flocculent, and not so thoroughly separated from the other elements of the fluid as the firm, hard curd of cow's milk is from the whey in which it floats." (West.)

When we reflect that the digestive organs of the *human* infant are found to digest human milk, and the force of its gastric juice proportioned to the solution of its soft, flocculent coagulum, we can understand why the solvent power of its gastric juice is sometimes unequal to redigesting the firm curd of cow's milk. When such is the case, acetous fermentation is quickly set up ; offensive gases distend the stomach and taint the breath, vomiting and diarrhœa set in, and in process of time the little patient sinks into a miserable state of marasmus, and dies. The remedy for this state of things is simple, for although we cannot change the elementary composition of the milk we have to use, we can introduce into the infant's stomach a digestive power proportioned to the food it has to use—the organic principle of digestion taken from the stomach of the calf.

It is now many years since I first applied this simple theory to practice in the case of one of my own children, who, when about three or four months old, was reduced to a condition of marasmus by vomiting and diarrhœa due to imperfect digestion of cow's milk. I ordered him fifteen or twenty drops of Pepsine wine, to be given immediately before or after each meal. Soon after commencing it he began to improve, and by degrees all bad symptoms vanished, and nutrition was quite restored.

The Pepsine was continued until he was nearly two years old, and he throve at least as well as if he had been wet-nursed ; other treatment, of course, pre-aided and accompanied the use of Pepsine, but was not until the latter was commenced that improvement took place.

Shortly after, a child born in England, and bottle-fed, was brought over to this country when about six months old; he also was suffering from infantile dyspepsia, and was pining away in a listless, apathetic state, quite indifferent to surrounding objects, and appearing as if he would lapse into idiocy from mal-nutrition of the nervous centres. I immediately ordered him Pepsine wine, which produced such beneficial effects, that after it had been continued about twelve months, he had become a bright, intelligent, well-nourished child.

Since then I have never recommended a wet-nurse, and have used Pepsine wine largely in dispensary, hospital and private practice, and have seen many apparently hopeless cases recover under its use.—*Dublin Journal of Medical Science, February.*

